# Fine-Scale Environmental Heterogeneity Drives Intra- and Inter-Site Variation in *Taraxacum officinale* Flowering Phenology

**DOI:** 10.3390/plants14142211

**Published:** 2025-07-17

**Authors:** Myung-Hyun Kim, Young-Ju Oh

**Affiliations:** 1 R&D Planning Division, Rural Development Administration, Jeonju 54875, Republic of Korea; 2 Institute for Future Environmental Ecology Co., Ltd., Jeonju 54883, Republic of Korea; cave50joo@gmail.com

**Keywords:** climate change, flowering phenology, microenvironment, nonlinear modeling, spatial variation, *Taraxacum officinale*

## Abstract

Understanding how flowering phenology varies across spatial scales is essential for assessing plant responses to environmental heterogeneity under climate change. In this study, we investigated the flowering phenology of the plant species *Taraxacum officinale* across five sites in an agricultural region of Wanju, Republic of Korea. Each site contained five 1 m × 1 m quadrats, where the number of flowering heads was recorded at 1- to 2-day intervals during the spring flowering period (February to May). We applied the nlstimedist package in R to model flowering distributions and to estimate key phenological metrics including flowering onset (5%), peak (50%), and end (95%). The results revealed substantial variation in flowering timing and duration at both the intra-site (quadrat-level) and inter-site (site-level) scales. Across all sites, the mean onset, peak, end, and duration of flowering were day of year (DOY) 89.6, 101.5, 117.6, and 28.0, respectively. Although flowering onset showed relatively small variation across sites (DOY 88 to 92), flowering peak (DOY 97 to 108) and end dates (DOY 105 to 128) exhibited larger differences at the site level. Sites with dry soils and regularly mowed *Zoysia japonica* vegetation with minimal understory exhibited shorter flowering durations, while those with moist soils, complex microtopography, and diverse slope orientations showed delayed and prolonged flowering. These findings suggest that microhabitat variability—including landform type, slope direction, soil water content, and soil temperature—plays a key role in shaping local flowering dynamics. Recognizing this fine-scale heterogeneity is essential for improving phenological models and informing site-specific climate adaptation strategies.

## 1. Introduction

Climate change is fundamentally altering biological processes across ecosystems. Phenological shifts occur in plants (flowering, germination, and leaf senescence) and animals (migration, breeding, and emergence). These shifts serve as sensitive indicators of environmental change across taxa and latitudes [1,2,3,4,5,6,7,8,9,10,11]. Plant phenology is regulated by temperature, photoperiod, atmospheric CO_2_, precipitation, and soil moisture, serving as a reliable indicator of ecological responses to climate change [12,13,14].

Among the phenophases, flowering plays a pivotal role in reproductive success and in structuring biotic interactions such as pollination and herbivory [15,16,17,18,19]. Notably, a trend toward earlier flowering under global warming has been widely documented across various regions and time periods [3,5,20,21]. However, these shifts may result in mismatches in timing between interdependent species—such as between plants and their pollinators—potentially disrupting synchrony and compromising ecosystem stability [15,22,23].

While many flowering phenological studies have focused on broad-scale climatic gradients [12], recent research has emphasized the critical role of local-scale environmental heterogeneity. Variations in slope, soil moisture, light availability, and microtopography can induce substantial differences in flowering phenology even within the same climate zone [24,25,26,27]. Such fine-scale variation can mask patterns detected by coarse-resolution models, emphasizing the need for phenological analyses that explicitly incorporate microsite conditions [28].

Most phenological studies have focused on woody species in natural ecosystems during spring. In contrast, herbaceous species, anthropogenic habitats, and non-spring seasons are underrepresented [29]. This imbalance presents a critical gap in our understanding of plant phenological responses to climate change, particularly for species inhabiting disturbed environments such as agricultural fields, field margins, urban green spaces, and roadside verges.

The plant species *Taraxacum officinale* (common dandelion) is a cosmopolitan perennial herb that occurs in natural, agricultural, and urban habitats [30,31]. It is highly responsive to temperature and light cues and often flowers early in the growing season. These traits make it a particularly suitable model for studying fine-scale phenological variation and an effective indicator of local climatic conditions [32,33,34,35,36]. Its predominantly apomictic reproduction produces genetically uniform offspring, allowing environmental influences on phenology to be distinguished from genetic variation [37]. Additionally, its short generation time and visually distinct flowering heads facilitate repeated monitoring at multiple spatial scales. Owing to these characteristics, *T. officinale* has become a widely used species in citizen science phenology monitoring programs (e.g., USA National Phenology Network an UK Nature’s Calendar), which provide valuable data for tracking real-time biological responses to local climate variability [38,39].

Recent studies suggest that local environmental differences not only affect the timing of flowering but also the shape and duration of the flowering curve [40,41,42,43]. In this context, we employed a nested spatial framework to investigate how microenvironmental variation shapes flowering phenology under a uniform regional climate. We quantitatively evaluated how the onset, peak, and end of flowering, as well as the flowering duration of *T. officinale*, vary according to topography and habitat structure at two spatial scales—within populations (quadrat level) and among populations (site level). We aimed to elucidate how microenvironmental factors regulate flowering patterns and refine our understanding of spatial heterogeneity in plant phenology, thereby supporting climate adaptation strategies.

## 2. Results

### 2.1. Intra-Site Variation: Differences Among Quadrat-Level Phenology Within Sites

The nlstimedist model demonstrated an excellent fit to the flowering data across all quadrats, with the corrected residual sum squares (tdRSS) values exceeding 0.99 (Table S1), indicating highly reliable phenological parameter estimates.

Flowering phenology varied considerably among the five quadrats within each study site, indicating substantial intra-site heterogeneity (Table 1). Detailed flowering metrics and model parameter estimates (r, c, t) for each quadrat are provided in Table S1, supporting the phenological curve fits generated by the nlstimedist model.

Site 1 showed the greatest variability in flowering period, with a coefficient of variation (CV) of 44.74% and a range of 25.2 to 61.7 days. The end of flowering at Site 1 was also highly variable (CV = 8.98%; day of year [DOY] 120.1 to 145.4), suggesting the asynchronous termination of flowering among quadrats. This pronounced heterogeneity is shown in Figure S1, which displays CDF and PDF curves for each quadrat, illustrating differences in flowering timing and distribution shape.

In contrast, Site 3 showed minimal intra-site variation, with CVs for onset, peak, and end dates below 1%. The CVs for onset, peak, and end dates were only 0.74%, 0.51%, and 0.87%, respectively. The flowering duration was tightly clustered, ranging from 15.7 to 17.5 days (CV = 4.48%), indicating highly synchronized flowering dynamics among the quadrats within this site. These consistent flowering patterns, supported by uniform model parameters across quadrats, are depicted in Figure S3.

Site 4 also showed a relatively low variation in onset (CV = 1.75%) and peak flowering (CV = 0.99%), although flowering duration was more variable (CV = 11.99%), reflecting slight asynchrony in flower termination among quadrats within this site. This pattern is evident in the modeled curves in Figure S4, which show a minor variation in the right tails of the flowering distributions.

Site 2 presented moderate intra-site variability in flowering duration (CV = 15.07%) and end date (CV = 3.38%), despite a low variability in onset timing (CV = 2.04%). The variation in model parameters among quadrats at Site 2 is reflected in Figure S2, particularly in the spread and skew of the PDF curves.

Site 5 had a low variation in onset (CV = 1.25%) and peak (2.11%) dates, but displayed a substantial variability in both flowering end date (CV = 10.62%) and flowering duration (CV = 38.53%), with the flowering period ranging widely from 20.5 to 53.6 days among quadrats. These diverse flowering patterns are captured in the fitted curves in Figure S5, which illustrate divergence in termination timing and skewness based on model parameters.

Overall, the results reveal notable heterogeneity in flowering phenology at fine spatial scales. While certain sites (e.g., Sites 3 and 4) exhibited tight synchrony and consistent model fits among quadrats, others (e.g., Sites 1 and 5) displayed pronounced intra-site variation, particularly in flowering end dates and durations, as indicated by both observed metrics and model-derived parameters.

### 2.2. Inter-Site Variation: Phenological Differences Among Sites

Site-level flowering phenology was modeled by aggregating quadrat data and fitting the nlstimedist model, which achieved very high tdRSS values (above 0.99; Table S2), confirming the robustness of site-level phenological estimates. Spatial variation in flowering phenology across the five study sites was evaluated using two complementary approaches, as described in Section 4.3.2.

**Figure 1:**
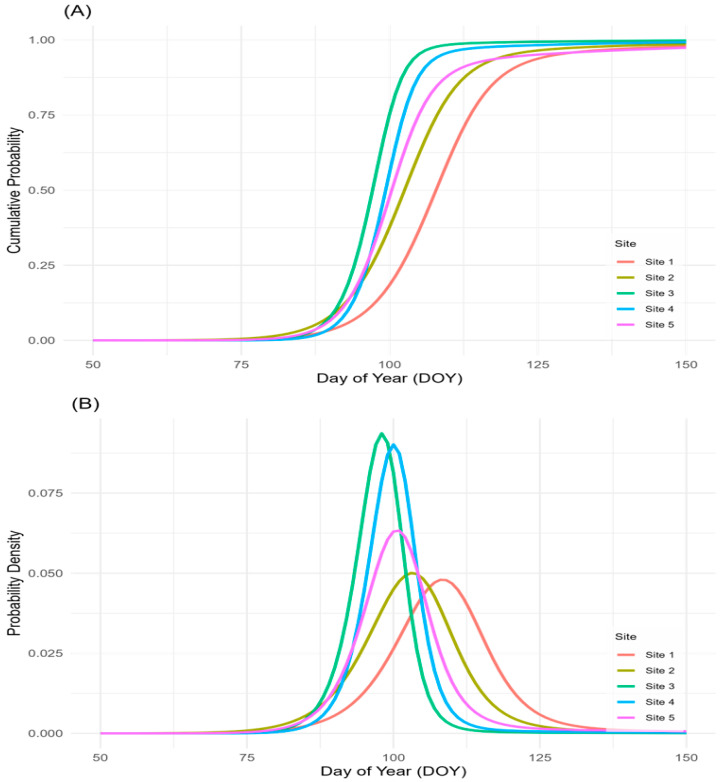
Site-level flowering distributions of *T. officinale* modeled using the nlstimedist package in R. (**A**) Cumulative distribution functions (CDFs) and (**B**) probability density functions (PDFs) are shown for each site. *X*-axis: day of year (DOY); *Y*-axis: cumulative flowering proportion (**A**) or flowering probability density (**B**). Curves are color-coded by site.

First, flowering head counts from all quadrats within each site were aggregated to create site-level time series data. These aggregated datasets were modeled using the *nlstimedist* package, which produced site-level cumulative distribution functions (CDFs) and probability density functions (PDFs) (Figure 1). From the fitted curves, key phenological metrics—onset, peak, end, and duration—were extracted for each site (Table 2), summarizing overall flowering dynamics at the site scale.

The onset of flowering ranged from day of year (DOY) 87.4 at Site 2 to 92.2 at Site 1, with a mean onset of 89.6 DOY (SD = 1.8; CV = 2.0%). Peak flowering occurred between DOY 97.2 (Site 3) and 107.9 (Site 1), with a mean of DOY 101.5 (SD = 3.7; CV = 3.6%). The end of flowering varied widely, from DOY 104.8 (Site 3) to 127.9 (Site 1), with a mean of DOY 117.6 (SD = 9.1; CV = 7.7%). Flowering duration exhibited the greatest variability among sites, ranging from 16.2 days (Site 3) to 36.9 days (Site 5), with a mean of 28.0 days (SD = 9.2; CV = 32.9%).

Across all five sites, the average flowering onset, peak, end, and duration were 89.6, 101.5, 117.6 DOY, and 28.0 days, respectively, representing the overall flowering pattern in this region. These global values serve as a baseline for comparing phenological variation across sites and can be used for further ecological or climate-response modeling.

Flowering phenology varied substantially across sites, reflecting the influence of local environmental conditions. Although onset dates differed by only 4.8 days (DOY 87.4–92.2), peak and end dates showed a wider divergence—10.7 days (DOY 97.2–107.9) and 23.1 days (DOY 104.8–127.9), respectively—suggesting increasing phenological separation as the season progressed. The duration of flowering ranged from 16.2 to 36.9 days, indicating that microclimatic and site-level factors shaped not only the timing but also the persistence of flowering.

Complete site-level flowering distributions along with estimated model parameters (r, c, and t) and additional curve descriptors (skewness, kurtosis, and entropy) are provided in Table S2. These parameters capture site-level differences in flowering intensity, spread, and symmetry, offering insights into the ecological drivers of phenological variation.

**Figure 2:**
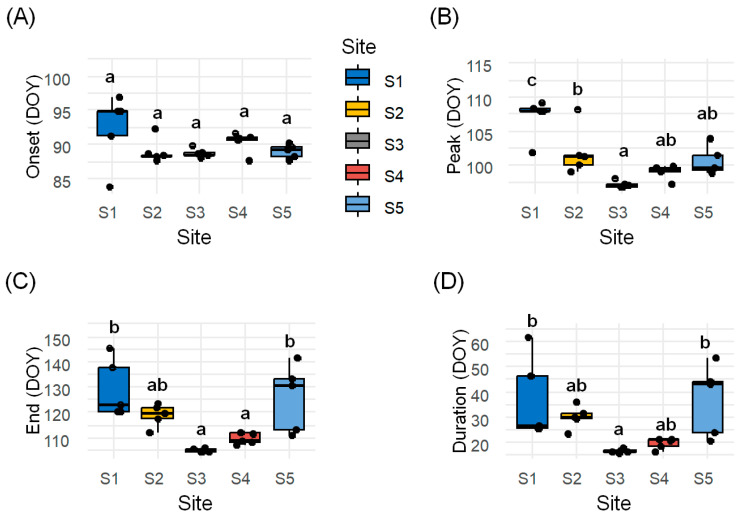
Comparison of flowering phenology metrics across five study sites. Boxplots show the (**A**) onset, (**B**) peak, (**C**) end, and (**D**) duration of flowering. Each point represents a quadrat-level estimate (n = 5 per site). Boxes indicate the interquartile range (IQR: 25th–75th percentiles); horizontal lines within boxes indicate the median; and whiskers extend to the minimum and maximum values within 1.5 × IQR. Different lowercase letters above boxes denote significant differences among sites (Tukey’s HSD, *p* < 0.05).

Second, to statistically assess phenological variation among sites while accounting for within-site variability, we treated quadrat-level phenology estimates as replicate observations nested within each site. We conducted one-way ANOVA tests for each phenological metric, followed by Tukey’s HSD tests for pairwise comparisons. Significant differences among sites were detected for peak flowering (*p* < 0.001), end of flowering (*p* < 0.001), and flowering duration (*p* < 0.01), while onset did not show statistically significant variation (*p* = 0.201). These results indicate that spatial environmental heterogeneity has a greater influence on the timing of peak and end flowering than on the onset of flowering, possibly due to site-specific differences in microclimate or resource availability. Figure 2 presents boxplots of each phenological metric across the five sites, with letters indicating statistically significant groupings based on Tukey’s HSD results.

In particular, flowering duration was the shortest at sites characterized by dry soil conditions and regularly mowed *Zoysia japonica* vegetation with minimal understory (e.g., Sites 3 and 4), and was the longest at sites with moist soils, complex microtopography, and mixed slope orientation (e.g., Sites 1 and 5). These environmental differences—including landform type, slope direction, soil water content, electrical conductivity (EC), and soil temperature—are likely key contributors to the observed site-level phenological variation.

Overall, these findings indicate that flowering onset in *T. officinale* is relatively stable across sites, whereas peak and end dates vary more substantially in response to fine-scale environmental and ecological differences.

## 3. Discussion

The excellent fit of the nlstimedist model (pseudo-R^2^ > 0.99; Tables S1 and S2) confirms the robustness of phenological parameter estimation across spatial scales, providing confidence in the patterns observed.

Among the phenological metrics, flowering onset exhibited the lowest coefficient of variation, indicating its relative stability as a phenological marker. In contrast, flowering peak and end dates showed higher variability, suggesting greater sensitivity to microhabitat conditions over time [44,45]. This pattern aligns with the skewness and kurtosis values derived from model fitting (Table S1), as well as with the ANOVA results; while onset did not differ significantly among sites (*p* = 0.20), peak, end, and duration differed significantly (*p* < 0.001, *p* < 0.01). Among the phenological metrics, flowering onset showed the least variation, confirming its relative temporal stability. In contrast, peak and end dates exhibited higher variability, suggesting greater sensitivity to local environmental conditions. This is consistent with the distribution shape metrics (skewness and kurtosis) and ANOVA results, where onset did not differ significantly among sites, unlike peak, end, and duration.

Our findings reveal that spatial variation in flowering onset, peak, and end dates is not only evident across sites but is also pronounced within sites, indicating that microhabitat heterogeneity significantly structures flowering dynamics in *T. officinale*.

As an early-flowering and temperature-sensitive species [32,33,36], *T. officinale* is widely used to detect phenological shifts in response to climate change. However, the observed differences in flowering phenology among the sites in this study appear more closely related to habitat environmental factors than broad climatic gradients. Habitat factors such as soil moisture, slope aspect, solar radiation, and vegetation type and maintenance showed substantial variation across sites and significantly influenced flowering and other aspects of plant phenology [46,47,48,49]. For instance, Sites 1 and 5, characterized by higher soil moisture and complex microtopography, exhibited later flowering timing and prolonged flowering duration. In contrast, Sites 3 and 4, characterized by dry conditions and *Zoysia japonica* lawns that were regularly mowed with sparse litter and understory vegetation, displayed earlier and shorter flowering, consistent with rapid reproductive strategies under environmental stress [50,51,52].

Intra-site variation among quadrats further supports the impact of microhabitat environments on flowering phenology. Such within-site variability may affect reproductive success and phenological synchrony with pollinators, playing an important role in community interactions and ecosystem functioning [6,8,40,41].

To evaluate the ecological significance of spatial variation, we reviewed prior studies reporting phenological shifts associated with climate warming (Table 3). For example, flowering onset typically advances by several days per decade or per 1 °C increase in temperature [19,34,40,42], and peak flowering shifts by about 5 days per decade in spring [40]. End dates and flowering duration show variable changes depending on species and regions [19,40]. In comparison, this study found that the maximum intra-site variation (i.e., among quadrats) in flowering onset reached 13.1 days, while inter-site variation was 4.8 days. These values are comparable to or exceed previously reported phenological shifts of 3.4 to 7.1 days per decade or per 1 °C temperature increase [19,34,40,42]. For peak flowering, we observed up to 9.3 days of variation within sites and 10.7 days among sites, which also falls within or exceeds the range of reported peak advances of approximately 3.3 to 5.3 days per decade under climate warming [40]. The end of flowering differed by 31.0 days within sites and by 23.1 days among sites, substantially exceeding the reported climate-driven advances in end dates of 2–3 days per decade [19,40]. Similarly, flowering duration varied by up to 36.5 days within sites and 20.7 days among sites, whereas reported climate-driven increases in flowering duration range from +2.6 to +8.9 days per decade [19,40]. These comparisons collectively highlight that fine-scale environmental heterogeneity can induce phenological variation that is comparable to or even greater than long-term trends associated with climate change. While some studies report both advances and delays in flowering end dates depending on species and region, this study focuses on the magnitude of variation rather than directionality. Species-specific and regional differences in phenological responses warrant further detailed investigation in future research.

This finding has important ecological implications. The fact that habitat heterogeneity leads to greater phenological variation than interannual climate variability suggests the potential for phenological mismatches between plants and their pollinators. Such mismatches may reduce pollination efficiency and reproductive success, thereby altering competitive interactions and affecting resource availability within plant communities [23,53]. As shown in Figure S6, distinct environmental gradients across the five study sites were observed. For example, Site 5 exhibited a significantly higher soil water content, while Site 4 showed the highest soil temperature. Sites 3 and 4, which had dry soils and were dominated by regularly mowed *Z. japonica* with minimal understory, exhibited earlier flowering onset and the shortest flowering durations. In contrast, Sites 1 and 5 featured moist soils, complex topography, and mixed slope directions, leading to delayed and prolonged flowering. These patterns highlight that landform type, slope direction, soil moisture, EC, and soil temperature are primary microenvironmental drivers of flowering phenology in *T. officinale* under uniform regional climate conditions. While the environmental measurements enabled us to identify broad relationships between site conditions and phenological patterns, we did not apply multivariate statistical models, as these environmental data were collected only once and the number of sites was insufficient for robust regression analysis. Instead, we interpreted site-level phenological variation in relation to relative environmental differences, supported by statistical comparisons and visual summaries (e.g., Table 4, as well as Figure 1 and Figure 2). These findings reinforce the interpretation that soil temperature and moisture are primary drivers of *T. officinale* flowering phenology and demonstrate that fine-scale habitat heterogeneity can induce substantial phenological variation. Future studies incorporating repeated environmental measurements and greater site replication would be valuable for modeling the relative contributions of these drivers more precisely.

Overall, our results underscore the importance of incorporating fine-scale environmental heterogeneity into phenological studies. Recognizing spatial drivers of flowering variation improves the forecasting of species’ climate responses [54,55]. Such knowledge may also guide localized phenological monitoring systems aimed at biodiversity conservation [56]. This fine-scale temporal mismatch in flowering among microhabitats may disrupt synchrony between plants and their pollinators, especially for generalist pollinators that depend on predictable floral resources. Asynchronous flowering can lead to decreased pollinator visitation rates within certain quadrats or sites, thereby reducing the likelihood of successful cross-pollination. Over time, such spatially inconsistent pollination may lower seed set, affect population dynamics, and shift plant–pollinator network structures [23,53].

## 4. Materials and Methods

### 4.1. Study Area and Experimental Design

**Figure 3:**
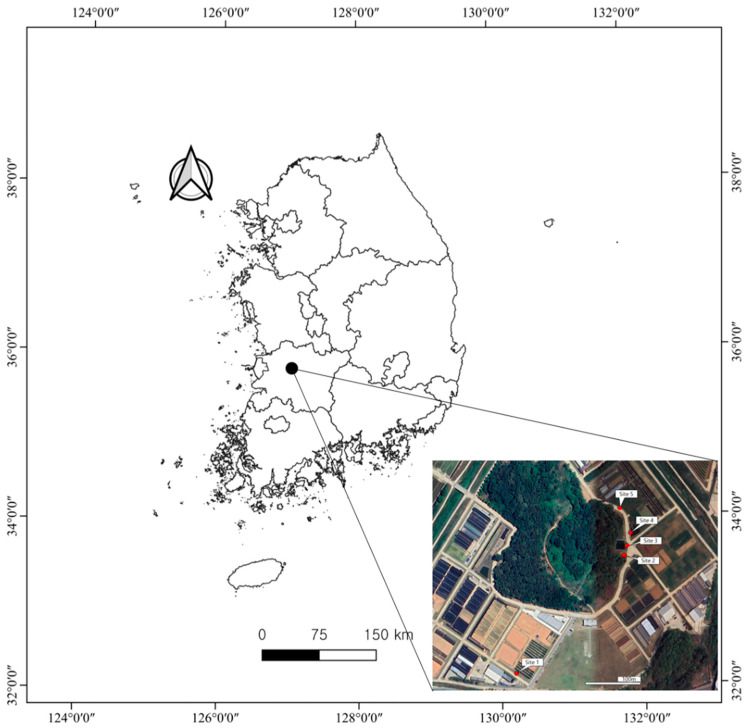
Map showing the survey region in Wanju, Republic of Korea, and a magnified view indicating the locations of the five survey sites.

**Figure 4:**
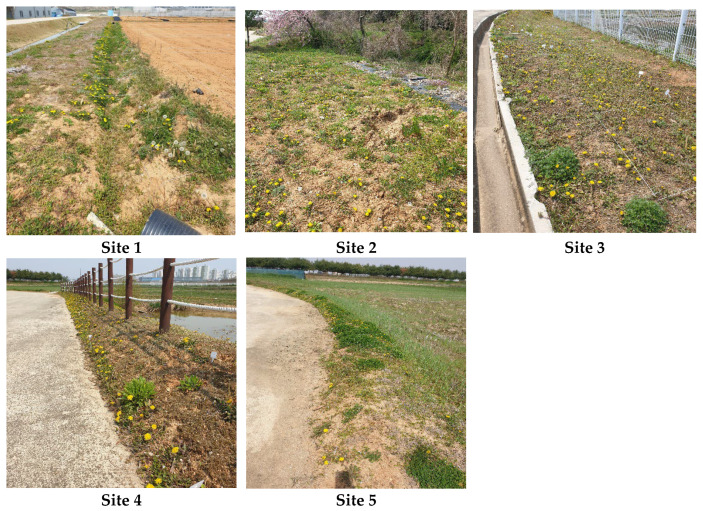
Field photographs illustrating environmental conditions at each of the five study sites (Sites 1–5).

The field survey was conducted in experimental agricultural fields located within the premises of the National Institute of Agricultural Sciences (NAS) in Wanju, Jeollabuk-do Province, Republic of Korea (Figure 3). To assess the flowering phenology of *T. officinale* under various microenvironmental conditions, we selected five field sites where local populations of *T. officinale* were abundant. These sites included an upland field ridge with a drainage ditch (Site 1), an upland field adjacent to a forest edge (Site 2), a maintained *Zoysia japonica* lawn near an artificial reservoir (Site 3), a maintained *Z. japonica* lawn on a roadside ridge (Site 4), and a paddy field ridge (Site 5) (Figure 4).

At each site, five quadrats (1 m × 1 m) were established, totaling 25 quadrats across all sites. The number of flowering heads in each quadrat was recorded during the spring flowering period. These quadrats served as the fundamental observational units to assess variation in flowering phenology across spatial scales, both intra-site (quadrat level) and inter-site (site level).

To assess the potential influence of microhabitat conditions on flowering phenology, soil water content, electrical conductivity (EC), and soil temperature were measured during the peak flowering period (mid-April) using a WT1000N sensor (MIRAE Sensor, Bucheon, Republic of Korea). These variables were selected based on previous studies that have highlighted their influence on flowering phenology across various plant taxa, including early-flowering and temperature-sensitive species [46,47,48,49]. At each site, measurements were taken at 15 randomly selected points to capture spatial variability in microenvironmental conditions. As these environmental variables were measured only once during the peak flowering period, they serve primarily as indicators of relative site conditions rather than time-series predictors. Therefore, we did not conduct multivariate or regression analysis, given the limited temporal resolution and small number of sites (n = 5). Instead, we used these site-level measurements to qualitatively interpret phenological patterns across the five locations.

### 4.2. Phenological Observation

*T. officinale*, a plant species widely distributed across North America, Europe, and Asia, occurs nationwide in Korea. It flowers year-round in temperate regions [32,57]. In Korea, it typically exhibits two flowering peaks—in spring and autumn. Among these, the spring flowering peak is much more pronounced in both abundance and frequency, whereas the autumn peak tends to be less consistent and generally smaller in magnitude [32].

To assess spatial variation in flowering phenology across multiple observational scales (quadrat and site), we focused on the primary spring flowering season. Field surveys were conducted from February to May in 2020, during which *T. officinale* displayed its active flowering. Observations were carried out at 1- or 2-day intervals to capture fine-scale temporal variation in flowering dynamics.

Accordingly, to investigate spatial variation in flowering phenology, this study focused on the primary flowering season in spring, when flowering activity is most intense. Field surveys were conducted from February to May 2020, during which *T. officinale* initiates active flowering and reproductive activity. To capture fine-scale temporal variation in flowering dynamics, observations were made at 1- or 2-day intervals. Surveys were carried out considering multiple spatial scales, including quadrats and sites.

### 4.3. Data Analysis

All statistical analyses were conducted using the R statistical environment (version 4.5.0) [58]. To evaluate spatial variation in flowering phenology, analyses were performed at two nested spatial scales—quadrat-level modeling (intra-site variation) and site-level modeling (inter-site comparison).

#### 4.3.1. Modeling Flowering Phenology Using Nlstimedist

Phenological processes such as flowering rarely occur at a constant rate over time. Although a simple exponential function could theoretically describe biological events that unfold uniformly [59], real-world phenological processes, in contrast, often exhibit nonlinear rates of change and delayed responses. To more accurately reflect these characteristics, the nlstimedist model embeds a lagged inverse-logit function within an exponential framework. This structure yields a biologically meaningful time-distribution function that effectively models unimodal phenological curves [59,60]. We applied this model to characterize the flowering phenology of *T. officinale*. The cumulative distribution function (CDF), describing the proportion of flowering events that have occurred by time x, is expressed as follows:

$$F(x)=1-{\left(1-\frac{r}{1+e^{-c\left(x-t\right)}}\right)}^{x}$$

The probability density function (PDF), representing the rate of flowering activity at time x, is the derivative of the CDF, as follows:

$$f\left(x\right)=\frac{dF(x)}{dx}={\left(1-\frac{r}{1+{\;e}^{-c\left(x-t\right)}}\right)}^{x}(\frac{{xrce}^{-c\left(x-t\right)}}{{\left(1+{\;e}^{-c\left(x-t\right)}\right)}^{2}\left(1-\frac{r}{1+e^{-c\left(x-t\right)}}\right)}-\ln\left(\frac{r}{1+e^{-c\left(x-t\right)}}\right))$$

Each parameter of the model has a clear biological interpretation. The parameter r is dimensionless and defines the maximum proportional rate at which flowering occurs. The parameter c (units: time^−1^) determines how rapidly r approaches its maximum value, shaping the steepness of the curve. The parameter t (units: time) represents the time-lag and reflects the delayed onset of the process following environmental cues. It can also be interpreted as a weighted timing metric, although it is not equivalent to a specific percentile.

Based on this model, we estimated biologically meaningful phenological metrics including the 5%, 50%, and 95% quantiles of the CDF, corresponding, respectively, to the onset, peak, and end of flowering. Flowering duration was calculated as the number of days between the 5% and 95% quantiles (i.e., end–onset). Additional distributional characteristics—such as mean, variance, skewness, kurtosis, and entropy—were also computed to assess the shape and complexity of the flowering curve. Model performance was evaluated using tdRSS.

#### 4.3.2. Quadrat-Level Modeling (Intra-Site Variation)

To examine spatial variation in flowering phenology within sites, we applied the above model independently to flowering time series data collected from individual quadrats. For each quadrat, the full set of phenological parameters—timing metrics (onset, peak, end, and duration), statistical moments, entropy, and tdRSS—was estimated.

To summarize intra-site variability, we computed descriptive statistics across quadrats within each site, including the mean, standard deviation, coefficient of variation (CV), and range of each metric. This allowed us to evaluate the extent of small-scale heterogeneity in flowering patterns.

#### 4.3.3. Site-Level Modeling (Inter-Site Variation)

To evaluate spatial variation in flowering phenology across sites, we employed two complementary approaches.

First, we aggregated the flowering head counts across all quadrats within each site to construct site-level time series datasets. These aggregated datasets were fitted using the same phenological model, allowing for a direct comparison of flowering patterns among sites in terms of onset, peak, end, and duration.

Second, we used the quadrat-level estimates obtained from Section 4.3.2 as replicate data points within each site. This enabled the statistical testing of inter-site differences using one-way ANOVA for each phenological metric. When significant site effects were detected, Tukey’s Honest Significant Difference (HSD) post hoc tests were performed to identify pairwise differences. All analyses were conducted in R version 4.5.0 (R Core Team, Vienna, Austria, 2025).

## 5. Conclusions

This study revealed substantial spatial variation in the flowering phenology of *T. officinale* within a climatically uniform region. By analyzing onset, peak, and end dates across five field sites and multiple quadrats per site, we demonstrated that local-scale environmental heterogeneity—such as soil moisture, slope direction, and microtopography—can significantly influence flowering dynamics.

To enhance predictive accuracy, future phenological models should incorporate microhabitat variability using high-resolution environmental data, including continuous soil moisture measurements, light availability, and fine-scale topography derived from remote sensing or UAV-based mapping. Integrating such data into process-based or machine learning models could better capture within-region differences and improve ecological forecasting.

Furthermore, these insights can inform land management and biodiversity conservation policies by identifying microhabitats vulnerable to phenological disruption, such as mismatches between flowering and pollinator activity. Establishing and maintaining localized plant–pollinator synchrony will be essential for ensuring ecosystem resilience. Developing localized phenological monitoring frameworks at fine spatial scales will support both scientific research and climate adaptation strategies.

## Figures and Tables

**Table 1:** Summary of flowering phenology metrics (onset, peak, and end dates in day of year and duration in days) for *Taraxacum officinale* across five study sites. Each value summarized five quadrats per site. Mean ± SD, coefficient of variation (CV), and observed range are shown for each metric.

Site	Onset (DOY)			Peak (DOY)			End (DOY)			Duration (Days)		
	Mean ± SD	CV (%)	Range	Mean ± SD	CV (%)	Range	Mean ± SD	CV (%)	Range	Mean ± SD	CV (%)	Range
S1	92.3 ± 5.2	5.64	83.7~96.8	107.1 ± 3.0	2.82	101.8~109.3	129.2 ± 11.6	8.98	120.1~145.4	36.9 ± 16.5	44.74	25.2~61.7
S2	89.0 ± 1.8	2.04	87.6~92.2	102.0 ± 3.7	3.58	99.0~108.3	118.9 ± 4.6	3.83	111.8~123.5	29.9 ± 4.5	15.07	23.2~35.8
S3	88.7 ± 0.7	0.74	88.1~89.8	97.2 ± 0.5	0.51	96.8~98.0	105.0 ± 0.9	0.87	104.1~106.3	16.3 ± 0.7	4.48	15.7~17.5
S4	90.4 ± 1.6	1.75	87.6~91.7	99.0 ± 1.0	0.99	97.3~99.9	109.7 ± 2.1	1.87	107.4~111.8	19.3 ± 2.3	11.99	15.8~21.1
S5	89.0 ± 1.1	1.25	87.5~90.3	100.7 ± 2.1	2.11	98.9~104.0	125 ± 13.4	10.62	110.8~141.8	37.0 ± 14.2	38.53	20.5~53.6

**Table 2:** Site-level flowering phenology metrics (onset, peak, end, and duration) of *T. officinale* at five field sites.

Site	Onset (DOY)	Peak (DOY)	End (DOY)	Duration (Days)
S1	92.2	107.9	127.9	35.7
S2	87.4	102.6	120.3	32.9
S3	88.6	97.2	104.8	16.2
S4	90.8	99.4	109.0	18.2
S5	88.9	100.5	125.8	36.9
Mean (SD)	89.6 (1.8)	101.5 (3.7)	117.6 (9.1)	28.0 (9.2)
CV (%)	2.0	3.6	7.7	32.9

**Table 3:** Comparison of flowering phenological variation in this study with previously reported temporal shifts associated with climate warming across various regions.

Flowering Phenology	Maximum Intra-Site Difference (Days)	Maximum Inter-Site Difference (Days)	CaraDonna et al., 2014 (Days/Decade) [40]	Szabó et al., 2016 (Days/Decade) [34]	Lorer et al., 2023 (Days/°C) [42]	Pareja-Bonilla et al., 2025 (Days/Decade) [19]
Onset	13.1	4.8	−6.4	−3.4~−3.9	−7.1	−4.5
Peak	9.3	10.7	−5.3 (spring) −3.3 (summer)	-	-	-
End	31.0	23.1	+3.1	-	-	−2.3
Duration	36.5	20.7	+8.9	-	-	+2.6
Number of species	1 (*T. officinale*)	1 (*T. officinale*)	60 species	1 (*T. officinale*)	10 herbaceous species	269 species
Region	South Korea	South Korea	Calorado Rocky Mountains, USA	Hungary	Belgium, France (experiment)	Spain (Mediterranean region)
Warming magnitude	-	-	+0.4 °C per decade (summer air temperature increase)	Observational regression	+3 °C experimental warming	Elevation/latitude gradient

Note: Negative values (−) represent advances in flowering phenology (e.g., earlier onset or peak), while positive values (+) indicate delays or extensions (e.g., later end or longer duration). Intra- and inter-site differences in this study were calculated based on observed flowering onset, peak, end, and duration of *Taraxacum officinale* across quadrats and sites. Comparative values from other studies reflect decadal or temperature-driven phenological shifts derived from observational or experimental warming data.

**Table 4:** Environmental characteristics of the five study sites, including landform type, slope direction, soil water content, electrical conductivity (EC), and soil temperature. Values are presented as mean ± standard deviation (SD) with coefficient of variation (CV) in parentheses. Different superscript letters within each column indicate statistically significant differences among sites (Tukey’s HSD test, *p* < 0.05).

Site	Landform Type	Slope Direction	Soil Water Content ± SD (%)	Soil EC (ds/m)	Soil Temperature (℃)
Site 1	Upland field ridge with drainage ditch	Mixed (SW and EN)	20.8 ± 5.59 ^ab^ (CV = 26.9)	0.431 ± 0.040 ^ab^ (CV = 9.35)	22.9 ± 0.84 ^ab^ (CV = 03.68)
Site 2	Upland field adjacent to forest edge	Flat	22.5 ± 2.37 ^ab^ (CV = 10.6)	0.583 ± 0.062 ^c^ (CV = 10.50)	22.5 ± 2.18 ^a^ (CV = 9.66)
Site 3	Maintained law of *Zoysia japonica* near artificial reservoir	W	20.9 ± 1.40 ^ab^ (CV = 6.68)	0.383 ± 0.058 ^a^ (CV = 15.00)	24.1 ± 0.34 ^bc^ (CV = 1.42)
Site 4	Maintained law of *Zoysia japonica* on roadside ridge	W	19.7 ± 1.97 ^a^ (CV = 10.00)	0.457 ± 0.071 ^b^ (CV = 15.60)	24.3 ± 0.56 ^c^ (CV = 2.31)
Site 5	Paddy field ridge	Mixed (SW and EN)	24.0 ± 4.69 ^b^ (CV = 19.50)	0.463 ± 0.067 ^b^ (CV = 14.40)	24.2 ± 0.48 ^c^ (CV = 2.00)

Note: ^a b c^ Different letters within a column indicate significant differences among sites (*p* < 0.05, Tukey’s HSD). “Mixed” slopes indicate a combination of south–west (SW) and east–north (EN) directions.

## Data Availability

Some of the original data used for secondary analysis are included in the article. Additional data are available from the authors upon reasonable request.

## Supplementary Materials

The following supporting information can be downloaded at https://www.mdpi.com/article/10.3390/plants14142211/s1. Table S1: Quadrat-level flowering phenology and nlstimedist model parameters; Table S2: Site-level phenology and nlstimedist model parameters; Table S3: ANOVA summary for phenological metrics; Figures S1–S5: Site-wise CDFs and PDFs of flowering phenology; Figure S6: Environmental variability (boxplots).
