# Supplementary material for: Fine-Scale Environmental Heterogeneity Drives Intra- and Inter-Site Variation in Taraxacum officinale Flowering Phenology
Source: Plants (Basel). 2025 Jul 17;14(14):2211. doi: 10.3390/plants14142211 (PMC12300160; doi:10.3390/plants14142211)

Table S1. Flowering phenology metrics and nlstimedist model parameters for *Taraxacum officinale* observed in five quadrats at each of five sites. Phenology metrics include onset, peak, and end dates (day of year, DOY) and flowering duration (in days). Model parameters (r, c, t), tdRSS, skewness, kurtosis, and entropy were estimated using nonlinear curve fitting with the nlstimedist package in R.

Site	Quadrat	Flowering Phenology (DOY or days)				r	c	t	tdRSS	skewness	kurtosis	entropy
		Onset	Peak	End	Duration							
**S1**	**Q1**	83.7	101.8	145.4	61.7	0.020	0.153	106.3	0.992	4.43	31.70	5.92
	**Q2**	94.9	108.0	120.1	25.2	0.034	0.202	115.3	0.998	3.50	43.43	5.02
	**Q3**	91.3	108.4	137.6	46.3	0.022	0.160	114.1	0.997	4.73	38.76	5.72
	**Q4**	96.8	109.3	123.1	26.3	0.028	0.217	115.0	0.999	5.45	60.23	5.08
	**Q5**	94.9	108.0	120.1	25.2	0.034	0.202	115.3	0.998	3.50	43.42	5.02
	**Mean (SD)**	92.3	107.1	129.2	36.9	0.028	0.187	113.2	0.997	4.32	43.51	5.36
		(5.2)	(3.0)	(11.6)	(16.5)	(0.007)	(0.028)	(3.9)	(0.003)	(0.84)	(10.51)	(0.43)
**S2**	**Q1**	88.5	99.0	111.8	23.2	0.030	0.261	103.5	0.996	6.00	64.44	4.89
	**Q2**	87.6	100.0	123.5	35.8	0.024	0.223	104.2	0.994	5.44	47.25	5.29
	**Q3**	88.3	101.3	119.5	31.3	0.026	0.210	106.3	0.997	5.34	50.24	5.26
	**Q4**	88.2	101.5	117.7	29.5	0.028	0.205	107.0	0.996	5.11	50.77	5.22
	**Q5**	92.2	108.3	122.0	29.8	0.037	0.161	118.1	0.999	1.32	17.45	5.25
	**Mean (SD)**	89.0	102.0	118.9	29.9	0.029	0.212	107.8	0.996	4.44	46.63	5.18
		(1.8)	(3.7)	(4.6)	(4.5)	(0.005)	(0.037)	(6.0)	(0.002)	(1.84)	(16.88)	(0.16)
**S3**	**Q1**	88.4	97.1	104.5	16.1	0.043	0.307	102.3	0.997	4.00	61.96	4.37
	**Q2**	88.5	96.9	104.1	15.7	0.043	0.316	102.0	0.997	4.18	65.59	4.33
	**Q3**	88.9	97.2	106.3	17.5	0.033	0.330	101.1	0.996	6.90	84.61	4.51
	**Q4**	88.1	96.8	104.7	16.6	0.039	0.309	101.6	0.997	5.33	76.51	4.43
	**Q5**	89.8	98.0	105.6	15.8	0.039	0.326	102.6	0.995	5.87	86.07	4.37
	**Mean (SD)**	88.7	97.2	105.0	16.3	0.039	0.318	101.9	0.996	5.25	75.35	4.40
		(0.7)	(0.5)	(0.9)	(0.7)	(0.004)	(0.009)	(0.5)	(0.001)	(1.18)	(9.42)	(0.06)
**S4**	**Q1**	87.6	97.3	108.3	20.7	0.032	0.280	101.8	0.994	6.14	70.68	4.74
	**Q2**	90.7	99.0	111.8	21.1	0.027	0.342	102.0	0.995	6.76	69.47	4.64
	**Q3**	91.7	99.6	107.4	15.8	0.035	0.344	103.6	0.997	7.05	96.89	4.37
	**Q4**	90.8	99.3	108.9	18.0	0.031	0.324	103.2	0.997	6.92	83.51	4.55
	**Q5**	91.0	99.9	111.8	20.9	0.028	0.312	103.5	0.990	6.68	71.79	4.70
	**Mean (SD)**	90.4	99.0	109.7	19.3	0.031	0.312	102.8	0.995	6.71	78.47	4.60
		(1.6)	(1.0)	(1.9)	(2.3)	(0.003)	(0.025)	(0.9)	(0.002)	(0.34)	(11.16)	(0.14)
**S5**	**Q1**	89.6	99.7	113.3	23.7	0.028	0.273	103.8	0.997	6.21	64.29	4.88
	**Q2**	90.3	98.9	110.8	20.5	0.029	0.321	102.4	0.992	6.74	72.13	4.67
	**Q3**	89.2	99.3	133.4	44.2	0.022	0.279	102.2	0.993	5.54	44.54	5.17
	**Q4**	88.2	101.6	141.8	53.6	0.021	0.210	105.1	0.988	5.04	38.19	5.55
	**Q5**	87.5	104.0	130.5	43.0	0.023	0.166	109.6	0.990	4.70	39.10	5.65
	**Mean (SD)**	89.0	100.7	125.9	37.0	0.025	0.250	104.6	0.992	5.24	51.45	5.38
		(1.1)	(2.1)	(13.4)	(14.2)	(0.004)	(0.058)	(3.0)	(0.004)	(0.72)	(14.93)	(0.43)

Table S2. Flowering phenology metrics and nlstimedist model parameters for *T. officinale* observed in five sites. Phenology metrics include onset, peak, and end dates (day of year, DOY) and flowering duration (in days). Model parameters (r, c, t), tdRSS, skewness, kurtosis, and entropy were estimated using nonlinear curve fitting with the nlstimedist package in R.

Site	Flowering Phenology (DOY or days)				r	c	t	tdRSS	skewness	kurtosis	entropy
	Onset	Peak	End	Duration							
**Site 1**	92.2	107.9	127.9	35.7	0.025	0.173	114.1	0.999	4.79	44.64	5.48
**Site 2**	87.4	102.6	120.3	32.9	0.028	0.177	109.0	0.997	4.47	43.13	5.39

**Site 3**	88.6	97.2	104.8	16.2	0.040	0.314	102.0	0.998	5.24	77.10	4.40
**Site 4**	90.8	99.4	109.0	18.2	0.032	0.319	103.4	0.997	6.86	83.16	4.56
**Site 5**	88.9	100.5	125.8	36.9	0.024	0.242	104.1	0.995	5.58	47.91	5.23
**Mean (SD)**	89.6 (1.8)	101.5 (3.7)	117.6 (9.1)	28.0 (9.2)	0.030 (0.007)	0.245 (0.066)	106.5 (4.6)	0.997 (0.001)	5.39 (0.79)	59.59 (17.41)	5.01 (0.47)

Table S3. Summary of flowering phenology metrics (mean ± SD) for *T. officinale* across five study sites.

Flowering Phenology	Site 1	Site 2	Site 3	Site 4	Site 5	ANOVA *p*-value
**Onset**	92.3 ± 4.1	89.0 ± 2.0	88.7 ± 0.5	90.4 ± 1.5	89.0 ± 1.2	0.201
**Peak**	107.1 ± 1.9	102.0 ± 3.5	97.2 ± 0.5	99.0 ± 1.0	100.7 ± 2.0	**<0.001**
**End**	129.0 ± 9.0	119.0 ± 4.6	105.0 ± 0.9	110.0 ± 1.5	126.0 ± 8.4	**<0.001**
**Duration**	36.9 ± 13.2	29.9 ± 5.0	16.3 ± 0.9	19.3 ± 2.1	37.0 ± 9.9	**<0.01**

† Values in bold indicate statistically significant differences (ANOVA).

Figure S1. Flowering phenology curves for five quadrats in Site 1. Estimated (A) cumulative distribution function (CDFs) and (B) probability density functions (PDFs) are shown.

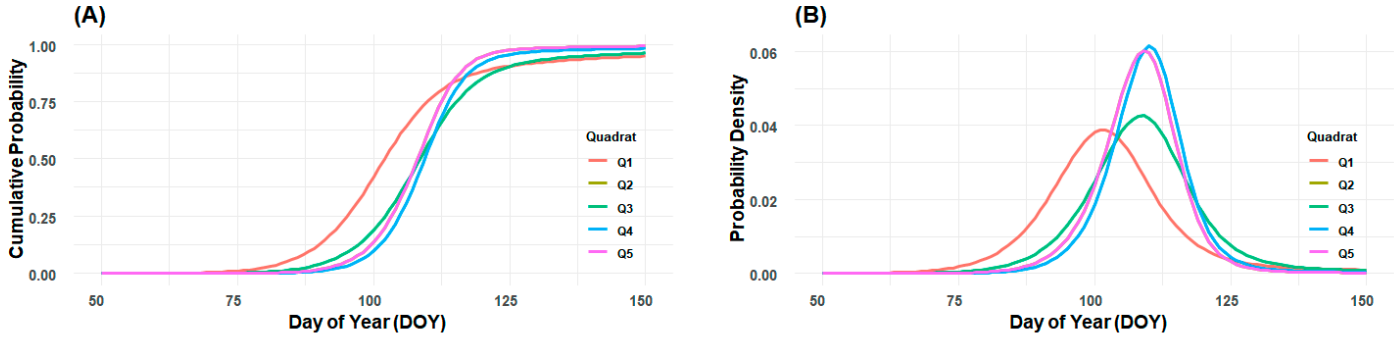

Figure S2. Flowering phenology curves for five quadrats in Site 2. Estimated (A) cumulative distribution function (CDFs) and (B) probability density functions (PDFs) are shown.

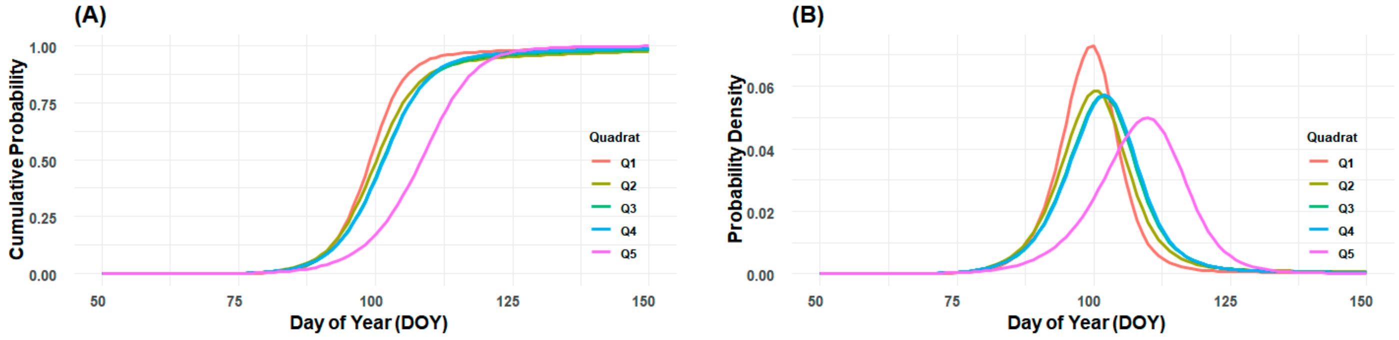

Figure S3. Flowering phenology curves for five quadrats in Site 3. Estimated (A) cumulative distribution function (CDFs) and (B) probability density functions (PDFs) are shown.

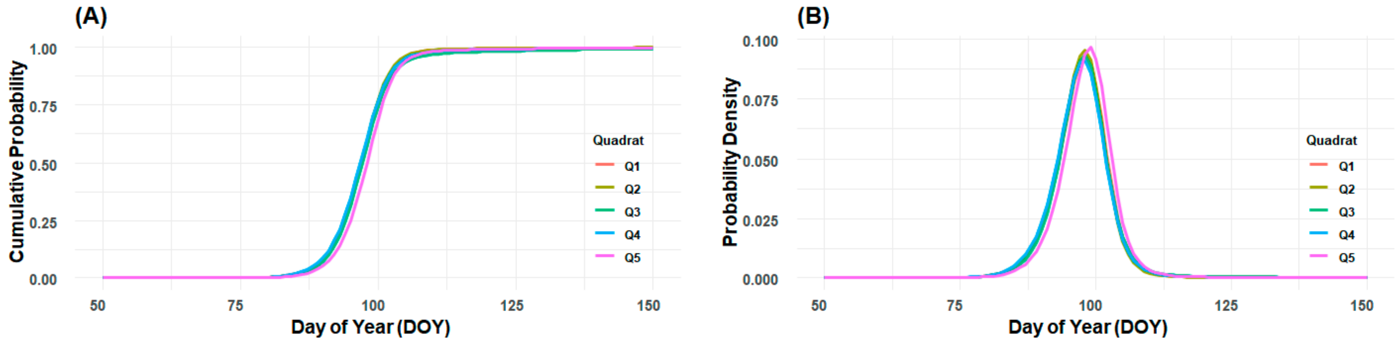

Figure S4. Flowering phenology curves for five quadrats in Site 4. Estimated (A) cumulative distribution function (CDFs) and (B)probability density functions (PDFs) are shown.

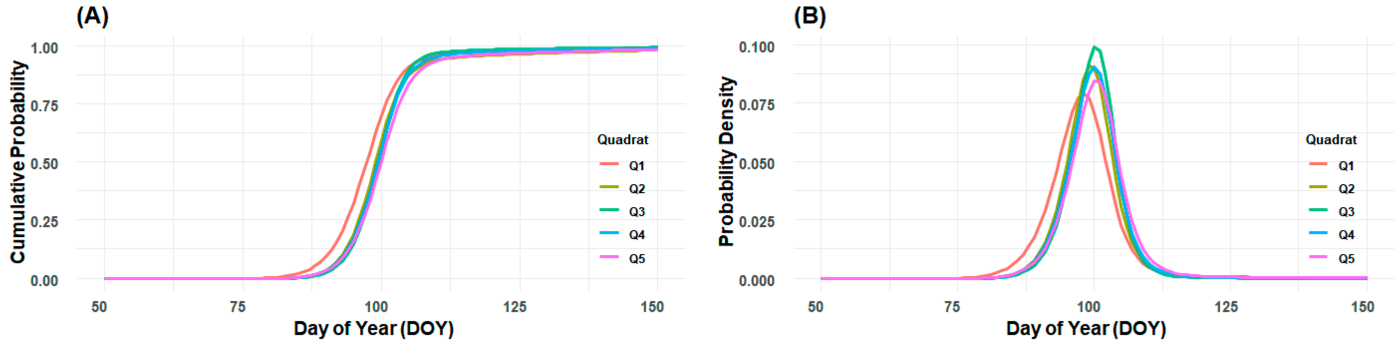

Figure S5. Flowering phenology curves for five quadrats in Site 5. Estimated (A) cumulative distribution function (CDFs) and (B) probability density functions (PDFs) are shown.

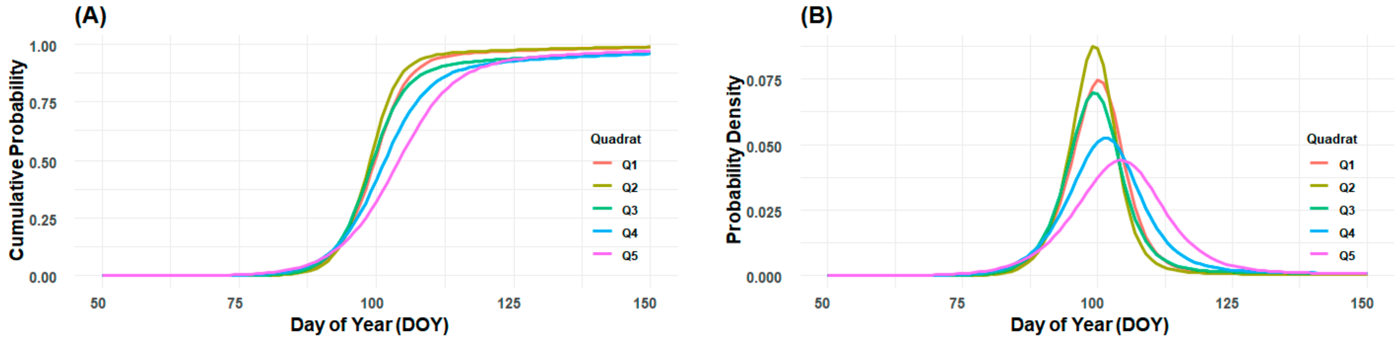

Figure S6. Boxplots showing variation in environmental conditions across five study sites.

(A) Soil water content, (B) electrical conductivity (EC), and (C) soil temperature. Boxes represent the interquartile range (IQR), with median lines and whiskers extending to 1.5× IQR. Different lowercase letters indicate significant differences among sites based on one-way ANOVA followed by Tukey's honest significant difference test (α = 0.05). Sites not sharing a letter differ significantly.

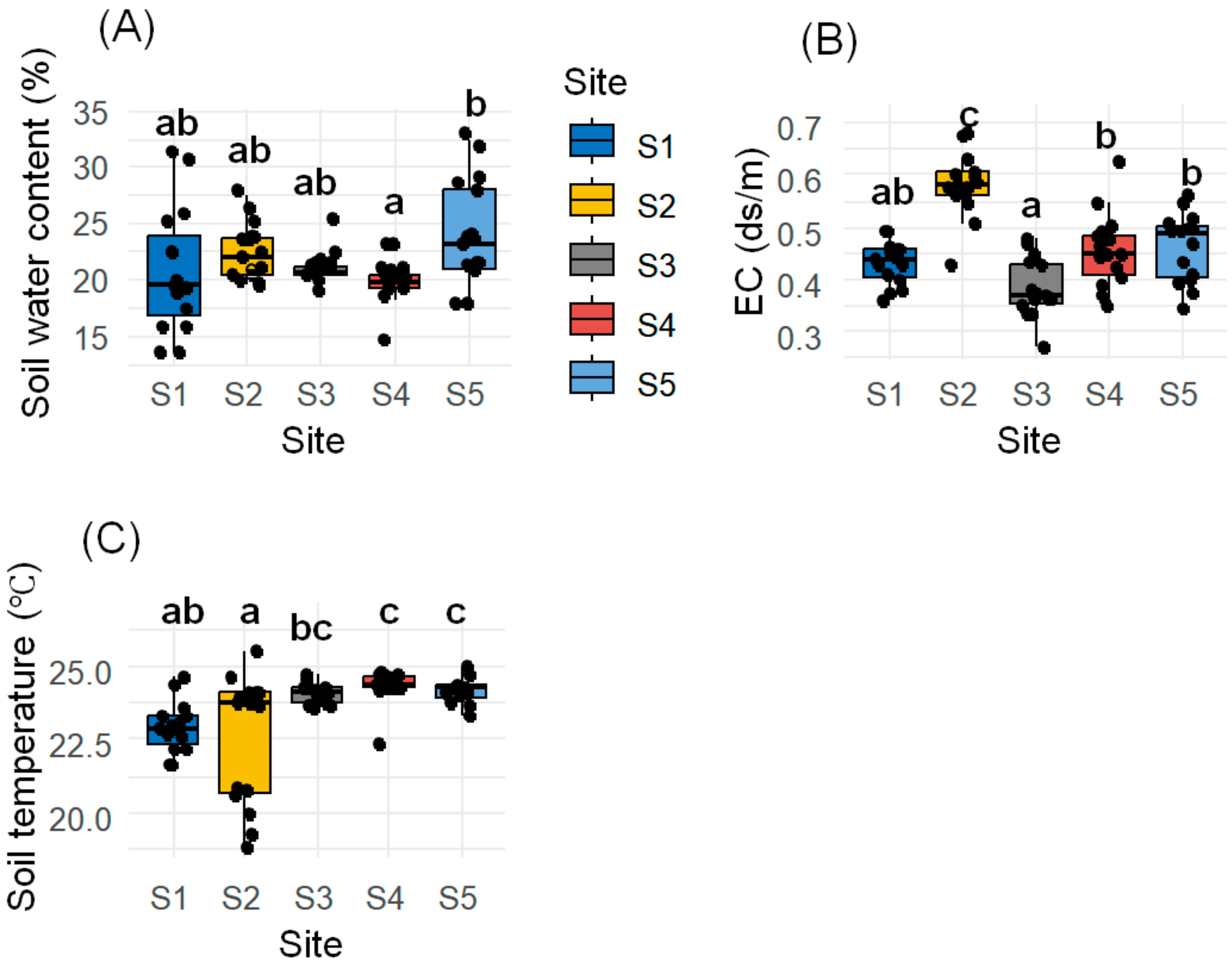

Supplement: Supplementary file 1 [file plants-14-02211-s001.zip › plants-3746994-supplementary.pdf]
